# Effects of head modeling errors on the spatial frequency representation of MEG

**DOI:** 10.1088/1361-6560/accc06

**Published:** 2023-04-27

**Authors:** Wan-Jin Yeo, Eric Larson, Joonas Iivanainen, Amir Borna, Jim McKay, Julia M Stephen, Peter D D Schwindt, Samu Taulu

**Affiliations:** 1 Department of Physics, University of Washington, Seattle, WA 98195, United States of America; 2 Institute for Learning and Brain Sciences, University of Washington, Seattle, WA 98195, United States of America; 3 Sandia National Laboratories, Albuquerque, NM 87123, United States of America; 4 Candoo Systems Inc., Port Coquitlam, BC V3C 5M2, Canada; 5 The Mind Research Network a Division of Lovelace Biomedical Research Institute, Albuquerque, NM 87106, United States of America

**Keywords:** magnetoencephalography, optically-pumped magnetometers, boundary element method, source reconstruction, spatial frequencies, inverse models

## Abstract

**Objectives.:**

We aim to investigate the effects of head model inaccuracies on signal and source reconstruction accuracies for various sensor array distances to the head. This allows for the assessment of the importance of head modeling for next-generation magnetoencephalography (MEG) sensors, optically-pumped magnetometers (OPM).

**Approach.:**

A 1-shell boundary element method (BEM) spherical head model with 642 vertices of radius 9 cm and conductivity of 0.33 S m^−1^ was defined. The vertices were then randomly perturbed radially up to 2%, 4%, 6%, 8% and 10% of the radius. For each head perturbation case, the forward signal was calculated for dipolar sources located at 2 cm, 4 cm, 6 cm and 8 cm from the origin (center of the sphere), and for a 324 sensor array located at 10 cm to 15 cm from the origin. Equivalent current dipole (ECD) source localization was performed for each of these forward signals. The signal for each perturbed spherical head case was then analyzed in the spatial frequency domain, and the signal and ECD errors were quantified relative to the unperturbed case.

**Main results.:**

In the noiseless and high signal-to-noise ratio (SNR) case of approximately ⩾6 dB, inaccuracies in our spherical BEM head conductor models lead to increased signal and ECD inaccuracies when sensor arrays are placed closer to the head. This is true especially in the case of deep and superficial sources. In the noisy case however, the higher SNR for closer sensor arrays allows for an improved ECD fit and outweighs the effects of head geometry inaccuracies.

**Significance.:**

OPMs may be placed directly on the head, as opposed to the more commonly used superconducting quantum interference device sensors which must be placed a few centimeters away from the head. OPMs thus allow for signals of higher spatial resolution to be captured, resulting in potentially more accurate source localizations. Our results suggest that an increased emphasis on accurate head modeling for OPMs may be necessary to fully realize its improved source localization potential.

## 1. Introduction

Magnetoencephalography (MEG) is a non-invasive neuroimaging modality that provides spatiotemporal estimates of brain activity (Hämäläinen et al 1993, Hari and Puce 2017, Ilmoniemi and Sarvas 2019). These estimates are based on inverse modeling, i.e. inferring the distribution of electric current in brain tissue based on a measurement of the associated magnetic field. Until recently, the only practical sensor type sensitive enough for MEG measurements has been the superconducting quantum interference device (SQUID), which requires cryogenics in order to maintain the superconducting state of the sensors. The necessary thermal insulation between the sensors, which must be held in a dewar containing liquid helium, and the surface of the head creates a minimum gap of at least 2 cm between the scalp and the sensors. Reducing the measurement distance is imperative to further improve spatial resolution of MEG as the magnetic fields decay rapidly as a function of distance. Fortunately, recent developments in sensor technology, especially in the domain of optically-pumped magnetometers (OPM), have made on-scalp MEG possible (Knappe et al 2014, Borna et al 2017, Boto et al 2017, Iivanainen et al 2017, Borna et al 2020, Hill et al 2020). The reduced proximity between the sensors and the brain sources has the potential to significantly improve the spatial resolution of MEG, since the detected signal strength and spatial complexity are expected to increase.

Brain sources are typically modeled as having a primary current and volume current. The primary current is where neural activity occurs and can be modeled as a current dipole in many cases, especially for focal sources. The volume current is the passive return current that closes the current loop inside the head. The volume current can be represented as a corresponding (non-physical) surface current on the head boundaries that produces an equivalent magnetic field, assuming that the head is modeled as a closed, piecewise homogeneous conductor (Geselowitz 1967). Thus, any inaccuracies in head geometries that are involved in forward signal calculations will inaccurately account for the volume current, resulting in an incorrect forward model. One such forward calculation method is the boundary element method (BEM). BEM typically uses triangulated, decimated surface meshes for the head model so that the electric potential, which is implicitly defined and difficult to determine in the continuous case, may be more easily calculated through some assumptions, such as uniform conductivity within compartments (Phillips et al 2006, Stenroos et al 2007, Mäkinen et al 2020).

For OPMs that can detect signals with higher spatial resolutions due to their decreased sensor-to-array distance, higher spatial frequency contributions from primary and volume currents are expected to be detected. Here, we investigate the importance of a spherical head’s BEM triangle mesh accuracy in the forward model of MEG signals as a function of sensor array distances. The mesh inaccuracies are introduced via perturbing the mesh vertices. We also investigate which spatial frequencies suffer from the greatest errors as a function of sensor array distance. We used the simplest and most straightforward BEM methods, the constant collocation (CC) and linear collocation (LC) approaches, to illustrate this effect. The simplicity of CC BEM has the added advantage of being able to easily illustrate the general method one may use to find analytical forms of the errors, as shown in the Supplementary material. Then, we illustrate the effects of the inaccurate forward signals in equivalent current dipole (ECD) source localization fits. We show that in the noiseless case, increased signal error due to BEM errors result in less accurate source localization, especially for deep sources. However, in the presence of noise, the increased signal-to-noise ratio (SNR) due to closer sensor array distances to the head generally still improves the source localization.

We first give an overview of BEM used in MEG signal forward calculations, with emphasis on the CC approach. Then, we discuss our results for the effects of BEM head geometry inaccuracies on the signal vectors for varying sensor array and source distances. We also analyze the errors in the spatial frequency domain via a decomposition into the Signal Space Separation (SSS) basis with varying *l*-degree truncation choices. Finally, we performed ECD fits with the inaccurate forward calculated signals to determine their effects on source localization in both noiseless and noisy sensor cases.

## 2. Boundary element method overview

### 2.1. Geselowitz’ formula

Itis common practice in MEG and EEG modeling to express the total current as a superposition of two components, i.e.

(1) $$J\left(r^{\prime }\right)=J^{P}\left(r^{\prime }\right)+J^{v}\left(r^{\prime }\right),$$

where $J^{P}\left(r^{\prime }\right)$ represents the physiologically interesting primary current and $J^{v}\left(r^{\prime }\right)$ is the associated passive volume current component that completes the loop of electric current in the brain tissue. On the basis of the Coulomb force, the passive volume current is of the form

(2) $$J^{v}\left(r^{\prime }\right)=\sigma E=-\sigma \nabla V,$$

where *σ*, **E**, and *V* are the electric conductivity, the electric field, and the electric potential, respectively. The second equality in equation (2) is valid when the time derivative of the magnetic field **B** is insignificant, which is the case in MEG and EEG (Hämäläinen et al 1993). If we assume a piecewise homogeneous conductor head model with *N_S_* conductivity boundary surfaces, the magnetic field due to **J**(**r**′) is given by *Geselowitz’ formula* (Geselowitz 1967, Hämäläinen et al 1993)

(3) $$B(r)=B_{0}(r)+\frac{\mu_{0}}{4\pi }\sum_{l=1}^{N_{S}}\left(\sigma_{l}^{-}-\sigma_{l}^{+}\right)\;\int_{S_{l^{\prime }}}V\left(r^{\prime }\right)\frac{r-r^{\prime }}{{\left|r-r^{\prime }\right|}^{3}}\times d{S^{\prime }}_{l},$$

where

(4) $$B_{0}(r)=\frac{\mu_{0}}{4\pi }\int_{v^{\prime }}J^{P}\left(r^{\prime }\right)\times \frac{r-r^{\prime }}{{\left|r-r^{\prime }\right|}^{3}}dv^{\prime }$$

is the contribution by primary currents, *v*′ is the total head volume, and $\sigma_{l}^{-}$ and $\sigma_{l}^{+}$ denote conductivities of the inner and outer regions relative to *S_l_*. The second term on the right hand side of (3) is the contribution by volume currents, which we will denote as **B**_*vol*_. We need the electric potential *V* in order to calculate **B**_*vol*_ and hence the total magnetic field. Below we review the expressions to obtain discrete values of *V* on the boundaries that we may thus use to approximate and discretize **B**.

### 2.2. Discretization of the electric potential field

Here, we give an overview of the CC BEM approach. The LC requires only a slight modification, as will be pointed out. More comprehensive reviews may be found in (de Munck 1992, Ferguson etal 1994, Phillips et al 2006, Stenroos et al 2007, Stenroos 2009).

The electric potential *V*(**r**) for a field point **r** on the *k*^th^ surface *S_k_* is given intrinsically by Hämäläinen et al (1993), Phillips et al (2006)

(5) $$V(r)=V_{\infty }(r)-\frac{1}{2\pi }\sum_{l=1}^{N_{S}}\;\frac{\sigma_{l}^{-}-\sigma_{l}^{+}}{\sigma_{k}^{-}+\sigma_{k}^{+}}\int_{{S_{l}}^{\prime }}V\left(r^{\prime }\right)\frac{r-r^{\prime }}{{\left|r-r^{\prime }\right|}^{3}}\cdot d{S^{\prime }}_{l},$$

where

(6) $$V_{\infty }(r)=\frac{1}{2\pi \left(\sigma_{k}^{-}+\sigma_{k}^{+}\right)}\int_{v^{\prime }}J^{P}\left(r^{\prime }\right)\cdot \frac{r-r^{\prime }}{{\left|r-r^{\prime }\right|}^{3}}dv^{\prime }.$$

Like in the **B** field case, equation (6) describes the primary current contribution to the potential whereas the surface integral is the volume current contribution. The primary current contribution is easily obtained if we have prescribed **J**^*P*^. In particular, if we let **J**^*P*^ be a collection of current dipoles with positions represented by delta functions, the integral collapses to a simple form. For N dipoles $J_{n}^{P}=Q_{n}\delta \left(r-r_{n}\right)$ where n=1,…,N, we have

(7) $$V_{\infty }(r)=\frac{1}{2\pi \left(\sigma_{k}^{-}+\sigma_{k}^{+}\right)}\sum_{n=1}^{N}Q_{n}\cdot \frac{r-r_{n}}{{\left|r-r_{n}\right|}^{3}}.$$

As for the volume current contribution, we may discretize each surface *S_l_* into *N_l_* triangles. Then, (5) can be written as

(8) $$V(r)=V_{\infty }(r)-\frac{1}{2\pi }\sum_{l=1}^{N_{S}}\frac{\sigma_{l}^{-}-\sigma_{l}^{+}}{\sigma_{k}^{-}+\sigma_{k}^{+}}\sum_{m=1}^{N_{l}}\int_{\Delta_{l}^{m}}V\left(r^{\prime }\right)\frac{r-r^{\prime }}{{\left|r-r^{\prime }\right|}^{3}}\cdot dS_{\Delta_{l}^{m^{\prime }}},$$

where $\Delta_{l}^{m}$ is the *m*th triangle of surface *S_l_*.

If we have chosen a large enough *N_l_* such that the triangle areas are small, we may reasonably make some assumptions about the behavior of the potential *V* within each triangle. In turn, *V* can be estimated with some basis and weight functions defined relative to parameters of relevant triangles. The potential *V* can then be explicitly defined and solved for. Many such approximations exist, including using constant or linear basis with collocation or Galerkin weighting (Stenroos 2009). Higher-degree basis functions have been considered as well (Gençer and Tanzer 1999).

In this paper, we use BEM as a tool to calculate the errors of volume current contributions due to closer sensors. Since we are not actually concerned with the accuracy between different approximation methods but rather the overall behaviour of the resulting signal due to head geometry errors, the most straightforward approximations which are the CC and LC approaches suffice for our purposes. We present the CC case in the next section which assumes a constant potential within each triangle. The LC approach approximates the potential within each triangle as a linear function via an interpolation from the potentials at the three vertices; see de Munck (1992), Stenroos et al (2007), Stenroos (2009). The simplicity of the CC approach allows us to outline an analytical method one may use to find errors for small boundary perturbations as well (see the supplementary material).

### 2.3. Linearization of electric potentials

First, we may assume that triangles are small enough such that the potential is constant in each triangle, i.e. $V(r^{\prime })\approx V(c_{l}^{m})$ when $r^{\prime }\in \Delta_{l}^{m}$, where $c_{l}^{m}$ is the centroid of $\Delta_{l}^{m}$. This allows us to pull the potential term out of the integral, and we get

(9) $$\int_{\Delta_{l}^{m}}V\left(r^{\prime }\right)\frac{r-r^{\prime }}{{\left|r-r^{\prime }\right|}^{3}}\cdot dS_{\Delta_{l}^{m}}\approx V\left(c_{l}^{m}\right)\int_{\Delta_{l}^{m}}\frac{r-r^{\prime }}{{\left|r-r^{\prime }\right|}^{3}}\cdot dS_{\Delta_{l}^{m\prime }}.$$

Notice that the integral on the right hand side is now simply the solid angle spanned by the triangle $\Delta_{l}^{m}$ from the observation point **r**; let us denote it as Ω*_l_^m^*(**r**). If we let **r**_*i*0_ ≡ **r**_*i*_ – **r**, *i* = 1, 2, 3 be the three vertices of the triangle relative to **r**, and let *r*_*i*0_ be their lengths, we may equivalently express each Ω*_l_^m^* as (Oosterom and Strakee 1983)

(10) $$\Omega_{l}^{m}=2\;arctan\left[\frac{r_{10}\cdot \left(r_{20}\times r_{30}\right)}{r_{10}r_{20}r_{30}+\left(r_{10}\cdot r_{20}\right)r_{30}+\left(r_{30}\cdot r_{10}\right)r_{20}+\left(r_{20}\cdot r_{30}\right)r_{10}}\right].$$

Let **r** coincide with centroids of the triangles as well. Then, all the potential terms of (5) are discretized at the same locations and it can now be compactly written as

(11) $$V\left(c_{k}^{i}\right)=V_{\infty }\left(c_{k}^{i}\right)-\frac{1}{2\pi }\sum_{l=1}^{N_{S}}\sum_{m=1}^{N_{l}}\frac{\sigma_{l}^{-}-\sigma_{l}^{+}}{\sigma_{k}^{-}+\sigma_{k}^{+}}V\left(c_{l}^{m}\right)\Omega_{l}^{m}\left(c_{k}^{i}\right).$$

In matrix form, this looks like

(12) $$\left[\begin{array}{c} V_{1}\\ \vdots \\ V_{N_{S}} \end{array}\right]=\left[\begin{array}{c} V_{\infty ,1}\\ \vdots \\ V_{\infty ,N_{S}} \end{array}\right]+\left[\begin{array}{ccc} G_{1,1} & \cdots & G_{1,N_{S}}\\ \vdots & \ddots & \vdots \\ G_{N_{S},1} & \cdots & G_{N_{S},N_{S}} \end{array}\right]\left[\begin{array}{c} V_{1}\\ \vdots \\ V_{N_{S}} \end{array}\right],$$

(13) $$G_{k,l}^{i,m}=-\frac{1}{2\pi }\frac{\sigma_{l}^{-}-\sigma_{l}^{+}}{\sigma_{k}^{-}+\sigma_{k}^{+}}\Omega_{l}^{m}\left(c_{k}^{i}\right).$$

Note that the **G** matrix is dependent only on the geometry and conductivities of the conductor, and it is also the only term that depends on the boundary geometry. This means that for each different source configuration in the same head model, **G** needs to be calculated only once, whereas the primary current contribution **V**_∞_ needs to be recalculated.

### 2.4. Matrix deflation

If we write the matrix equation (12) as $V=V_{\infty }+GV$, then we have

(14) $$(𝕀-G)V=V_{\infty },$$

where 𝕀 is the identity matrix. It is seemingly straightforward to solve for **V** by taking the inverse of (𝕀 – **G**), but (𝕀 − **G**) is actually non-invertible since itis rank-deficient; the electric potential has infinite number of solutions since it is defined up to an additive constant. This manifests from the fact that the fundamental equation we are trying to solve is the Poisson equation within the head,

(15) $$\nabla \cdot (\sigma \nabla V)=\nabla \cdot J^{p},$$

with Neumann boundary condition at each boundary (normal current continuity)

(16) $$\sigma^{+}\nabla V\cdot n=\sigma^{-}\nabla V\cdot n,$$

where **n** is the outward-pointing unit surface normal. These equations are specified only up to the first derivative/gradient of *V*, hence *V* is defined up to a constant. Note that the singularity of (𝕀 − **G**) is inherent to the physics of the problem at hand, and deflation is required regardless of the discretization scheme.

We thus know that both **V** and **V** + *k***e**, where **e** = (1,1,…,1)^*T*^ and *k* is a nonzero constant, are solutions to the matrix equation. So, in addition to (14), we also have

(17) $$(𝕀-G)(V+ke)=V_{\infty }.$$

Subtracting (14) from this yields

(18) (𝕀−G)e=0

which indicates that (𝕀 – **G**) has a zero eigenvalue with associated eigenvector **e** ≠ **0**, i.e. it is indeed singular. Equivalently,

(19) Ge=e.

This means that **G** has a unit eigenvalue with corresponding eigenvector **e**. So, one way to avoid the singularity is to eliminate this unit eigenvalue; the standard way to do this is by *deflation*, as follows.

First, assume the unit eigenvalue of **G** has a multiplicity of 1 (Lynn and Timlake 1968). For any vector **a**, we need to find a vector **c** with constant entries (not all necessarily the same) such that

(20) $$c^{T}a=\left\{\begin{array}{ll} k & \text{if}\;a=ke\\ 0 & \text{otherwise}. \end{array}\right.$$

The first case imposes the condition of defining a reference potential in some way. For example, if we pick **c** to have all the same entries, then it means we let the sum of all potentials over all boundaries to be zero (Hämäläinen and Sarvas 1989). We may also pick just a few entries to be zero or nonzero, corresponding to a possibly more meaningful reference potential (e.g. Wilson terminals used in electrocardiogram (Fischer et al 2002)). The second case ensures that all eigenvalues of **G′** = (**G** – **ec**^*T*^) are equal to the eigenvalues of **G**, except for the unit eigenvalue which is replaced by zero. This ensures that (𝕀 – **G′**) is non-singular and hence invertible by condition (19). We may explicitly show this preservation of eigenvalues for **G′** as follows. Let λ and **v**_*e*_ be eigenvalues and corresponding eigenvectors of **G**. For **v**_*e*_ ≠ **e**

(21) $$G^{\prime }v_{e}=\left(G-{ec}^{T}\right)v_{e}=Gv_{e}-{ec}^{T}v_{e}=\lambda v_{e}-e0=\lambda v_{e},$$

hence eigenvalues are preserved. For **v**_*e*_ = *k***e**,

(22) $$G^{\prime }ke=\left(G-{ec}^{T}\right)ke=Gke-{ec}^{T}ke=ke-ek=0.$$

So, we are now able to solve for **V** with the invertible deflated (𝕀 – **G′**)

(23) $$V={\left(𝕀-G+{ec}^{T}\right)}^{-1}V_{\infty }.$$

### 2.5. Discretization of the magnetic field

We now have a set of discrete potential solutions at the centroids of the triangles. If we discretize the **B**_*vol*_ integral in an identical manner as the electric potential case above, it allows us to get an approximation of the magnetic field at arbitrary field locations **r** directly from (3). The magnetic field is given by

(24) $$B(r)\approx B_{0}(r)+\frac{\mu_{0}}{4\pi }\sum_{l=1}^{N_{S}}\left(\sigma_{l}^{-}-\sigma_{l}^{+}\right)\sum_{m=1}^{N_{l}}V\left(c_{l}^{m}\right)\Omega_{l}^{m}$$

where we have defined the ”vector solid angle”

(25) $$\Omega_{l}^{m}\equiv \int_{\Delta_{l}^{m}}\frac{r-r^{\prime }}{{\left|r-r^{\prime }\right|}^{3}}\times dS_{\Delta_{l}^{m\prime }}.$$

The evaluation of $\Omega_{l}^{m}$ has been done in de Munck (1992) via Stoke’s Theorem, and is given by

(26) $$\Omega_{l}^{m}=\sum_{i=1}^{3}\left(\gamma_{i-1}-\gamma_{i}\right)r_{i},$$

where

(27) $$\gamma_{i}\equiv -\frac{1}{\left|r_{i+1}-r_{i}\right|}\cdot ln\frac{r_{i}\left|r_{i+1}-r_{i}\right|+r_{i}\cdot \left(r_{i+1}-r_{i}\right)}{r_{i+1}\left|r_{i+1}-r_{i}\right|+r_{i+1}\cdot \left(r_{i+1}-r_{i}\right)},$$

and *i* = 1, 2, 3 correspond to the three vertices of triangle *m* on surface *S_l_*. Also note that **r**_4_ ≡ **r**_1_ and **r**_0_ ≡ **r**_3_.

This expression allows us to calculate the magnetic field easily, assuming useful indexing of vertices and triangles has been done in the process of surface discretization.

### 2.6. Calculation of magnetic flux signals

For forward calculation of the signal vectors, we calculate the magnetic flux through magnetometer pick-up loops. In reality, this pick-up loop setup corresponds only for SQUIDs; OPMs measure the volume integral of the magnetic field over a cylindrical sensing volume. However, we are interested primarily in how the reduced distance of OPM sensor arrays may affect the signal measured due to BEM head model errors, hence we do not consider volume integrals over the magnetic field here.

For *N* sensors, the magnetic field is discretized into *N* channel readings of magnetic flux. The vectorization of these readings into an *N* × 1 vector *ϕ* is defined as the *signal vector*. The *signal space* (or *signal*) is the *N*-dimensional vector space with elements being any possible signal vector. Within the context of BEM, the signal space may be defined as the space containing all possible signals one may obtain when doing a forward calculation using the triangulation of a perfectly accurate head model.

In the case of sensors measuring the magnetic flux through a surface specified by an area and a normal vector, the *j*th element of *ϕ* is given by

(28) $$\phi_{j}=\int_{S_{j}}B(r)\cdot dS_{j},$$

where *d***S**_*j*_ represents an infinitesimal surface element on the sensor surface with unit normal **n**_*j*_. The calculation of (28) is commonly done by cubature approximation over the sensor area (Abramowitz and Stegun 1964).

## 3. Representation of perturbations in the signal and source space

### 3.1. Additive perturbation of signal space

From the BEM steps above, we see that any inaccurate head mesh models will lead to inaccurate forward calculations of the magnetic flux, since they correspond to perturbed triangle vertices and centroids. This in turn leads to inaccurate calculations of the potential, magnetic field and magnetic flux signal vector. These errors may be written as an additive perturbation since they are random and independent of the unperturbed quantities,

(29) $$V^{\prime }=V+\delta V$$

(30) $$B^{\prime }=B+\delta B$$

(31) $$\phi^{\prime }=\phi +\delta \phi ,$$

where the perturbations to each element of the flux signal vector are given by

(32) $$\delta \phi_{j}=\int_{S_{j}}\delta B(r)\cdot dS_{j}.$$

Note that the BEM errors are only relevant to the forward modeling of the signal vectors; real recorded signal vectors by definition do not have BEM errors. In the context of (noiseless) BEM forward modeling, the goal is to set up a head model such that the calculated signal *ϕ′* is as close to the (noiseless) recorded/true data *ϕ* as possible. If information about the BEM geometry is perfect, then *ϕ′* = *ϕ* with *δϕ* = 0. We re-emphasize that the primary current contribution (4) does not depend on the head model by Geselowitz’s formula, thus all errors come from inaccurate volume contribution. In other words, *δ***B** = *δ***B**_*vol*_ and hence *δϕ* = *δϕ_vol_*.

In the Supplementary material, we present an analytical approach to calculate the first-order perturbation contributions of *δ***V** and *δ***B**. The subsequent calculation of the flux perturbation *δϕ* may be done numerically with cubature approximations (Abramowitz and Stegun 1964) or analytically (Yeo et al 2021).

### 3.2. Quantification of signal reconstruction errors with subspace angle

A compact metric for quantifying the difference between the recorded/reference data and modeled/perturbed data is the angle between the corresponding signal vectors *ϕ* and *ϕ′* respectively. The angle between a vector **y** and a subspace **Z** can be calculated with the general formula (Gunawan et al 2005)

(33) $$subspace(p,Z)=\theta =arccos\left(\frac{\left\|{Proj}_{Z}p\right\|}{\left\|p\right\|}\right),$$

where ${Proj}_{z}p$ refers to the orthogonal projection of p onto Z. If the subspace Z is a vector as well, for instance, in our case where Z=ϕ and $p=\phi^{\prime }$, then the formula reduces to the familiar $\theta =arccos\left(\phi^{T}\phi^{\prime }/\|\phi \|\left\|\phi^{\prime }\right\|\right)$, and the order of the arguments in the notation ‘subspace(⋅,⋅)’ is interchangeable.

If we decompose the signals into their different spatial frequency components via the SSS method (Taulu and Kajola 2005), then the angle for individual different spatial frequency components can be calculated as well. In the SSS formalism, signal vectors are decomposed into their *l* degree components via a vector spherical harmonic (VSH) expansion, and each spatial frequency component is conveniently characterized by an *l* degree component. Higher *l* degrees correspond to higher spatial frequencies, whereas lower *l* degrees correspond to lower spatial frequency signal components. Let **S** be the basis matrix containing the VSH modes, **S**_1:*L*_ be the first *l* = *L* degree portion of **S**, and **x**_1:*L*_ be the corresponding coefficients/multipole moments of **S**_1:*L*_. The subspace angle *θ_l_* specific to the cumulative spatial frequency bands from *l* = 1 to *l* = *L* is thus

(34) $$\theta_{1:L}=arccos\left(\frac{\left\|{Proj}_{\phi_{1:L}}{\phi_{1:L}}^{\prime }\right\|}{\left\|\phi_{1:L}^{\prime }\right\|}\right),$$

where

(35) $$\phi_{1:L}=S_{1:L}x_{1:L}$$

and

(36) $$\phi_{1:L}^{\prime }=S_{1:L}x_{1:L}^{\prime }.$$

Given a measured signal vector *ϕ*, the multipole moments **x**_1:*L*_ must first be estimated by taking the pseudoinverse of **S**_1:*L*_,

(37) $$x_{1:L}\approx S_{1:L}^{†}\phi .$$

Then, if required, individual *l* degree portions **x**_1:*L*_ may be obtained from **x**_1:*L*_. Note that to avoid aliasing in signal reconstruction, a high enough *l* degree truncation is required so that the high frequency components of the signal do not get projected inaccurately onto basis vectors corresponding to low frequencies. It was shown in Taulu and Kajola (2005) that a truncation at approximately *L* = 8 is sufficient to represent signal vectors; in this paper, we truncate at *L* = 12 in anticipation of close sensor array distances capturing signals of higher spatial complexities.

## 4. Results

### 4.1. Simulation setup

For our reference set-up, we used a simple 1-shell spherical head model of radius 9 cm and conductivity 0.33 S m^−1^ with origin located at the center of the sphere, and did a CC BEM forward calculation as described in sections 2 and 3 to obtain *ϕ*. The BEM method was implemented using the Matlab library as provided in Stenroos et al (2007). The spherical surface was triangulated with 642 vertices, and 4 dipole sources with varying distances located at (2, 0, 0) cm, (4, 0, 0) cm, (6, 0, 0) cm, and (8, 0, 0) cm from the origin were specified. All the dipoles had a moment of (0, 10, 0) nAm. To simulate inaccurate mesh modeling to obtain *ϕ′*, the vertices of the spherical mesh were randomly perturbed radially up to 2%, 4%, 6%, 8% and 10% relative to the 9 cm radius.

**Figure 1.**
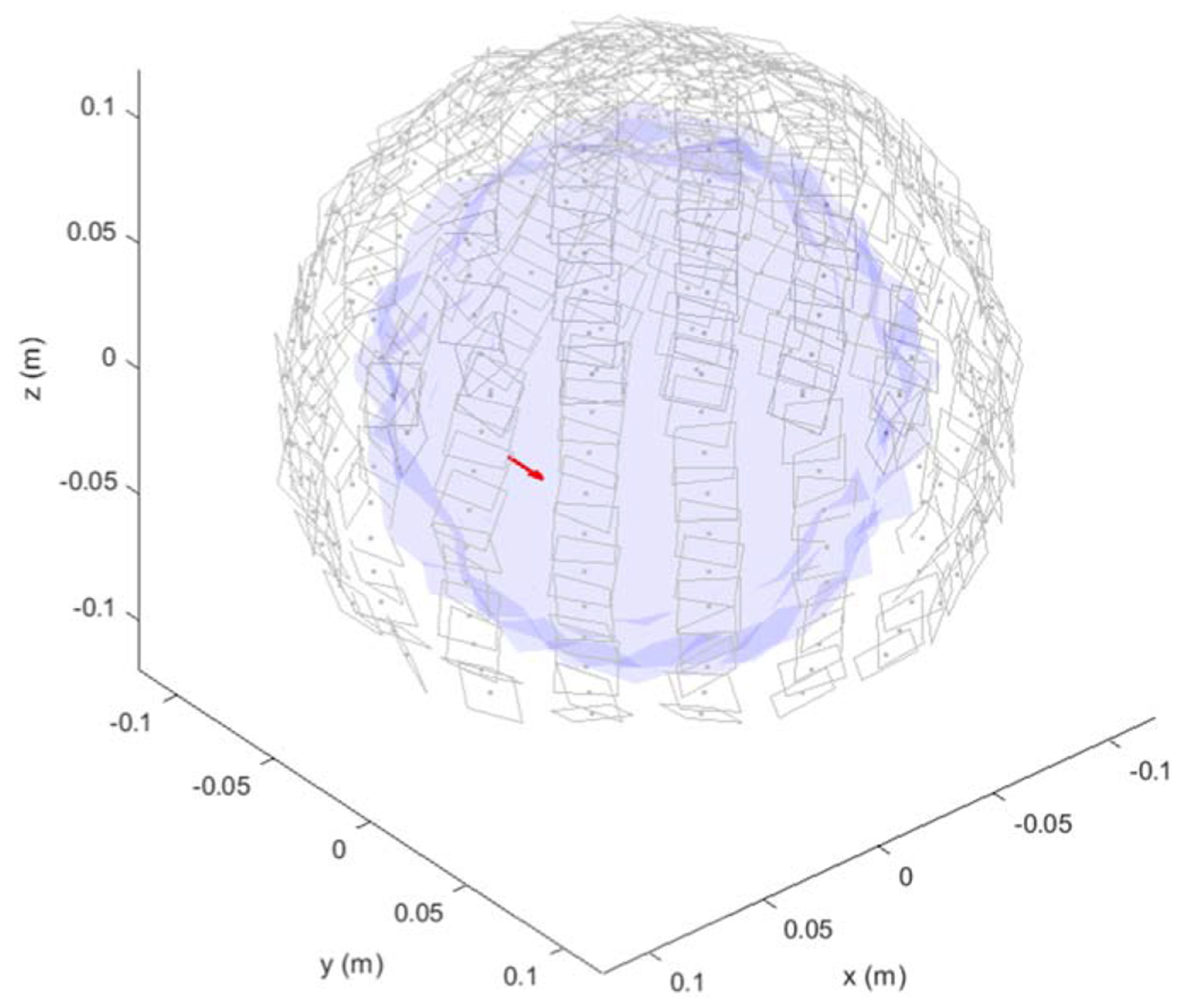
A (6, 0, 0) cm dipolar source with moment (0, 10, 0) nAm (red arrow) is shown located within a triangulated sphere of radius 9 cm that has its vertices randomly perturbed by up to 10% (blue mesh). The spherical sensor array of radius 10 cm with 324 square pick-up loops of side length 2.1 cm and random orientations is also shown in gray.

The sensor array that we used consisted of 324 square magnetometer pick-up loops with side length 2.1 cm all with non-radial orientations (to avoid linear dependence of the SSS basis (Taulu and Kajola 2005)), uniformly arranged on a spherical shell up to *π*/6 below the *z* = 0 plane. It has been shown in Yeo et al (2021) that a 9-point cubature approximation yields accurate calculations for the sensor distances of 10 cm to 15 cm that we considered, thus we used a 9-point cubature approximation for the calculation of signal vectors in this paper. Figure 1 illustrates the sensor, head, and source setup as described above.

The averaged results over 100 forward calculations with this random vertex perturbation setup is presented in the following sections.

### 4.2. Signal vector error for sensor arrays at varying distances

**Figure 2.**
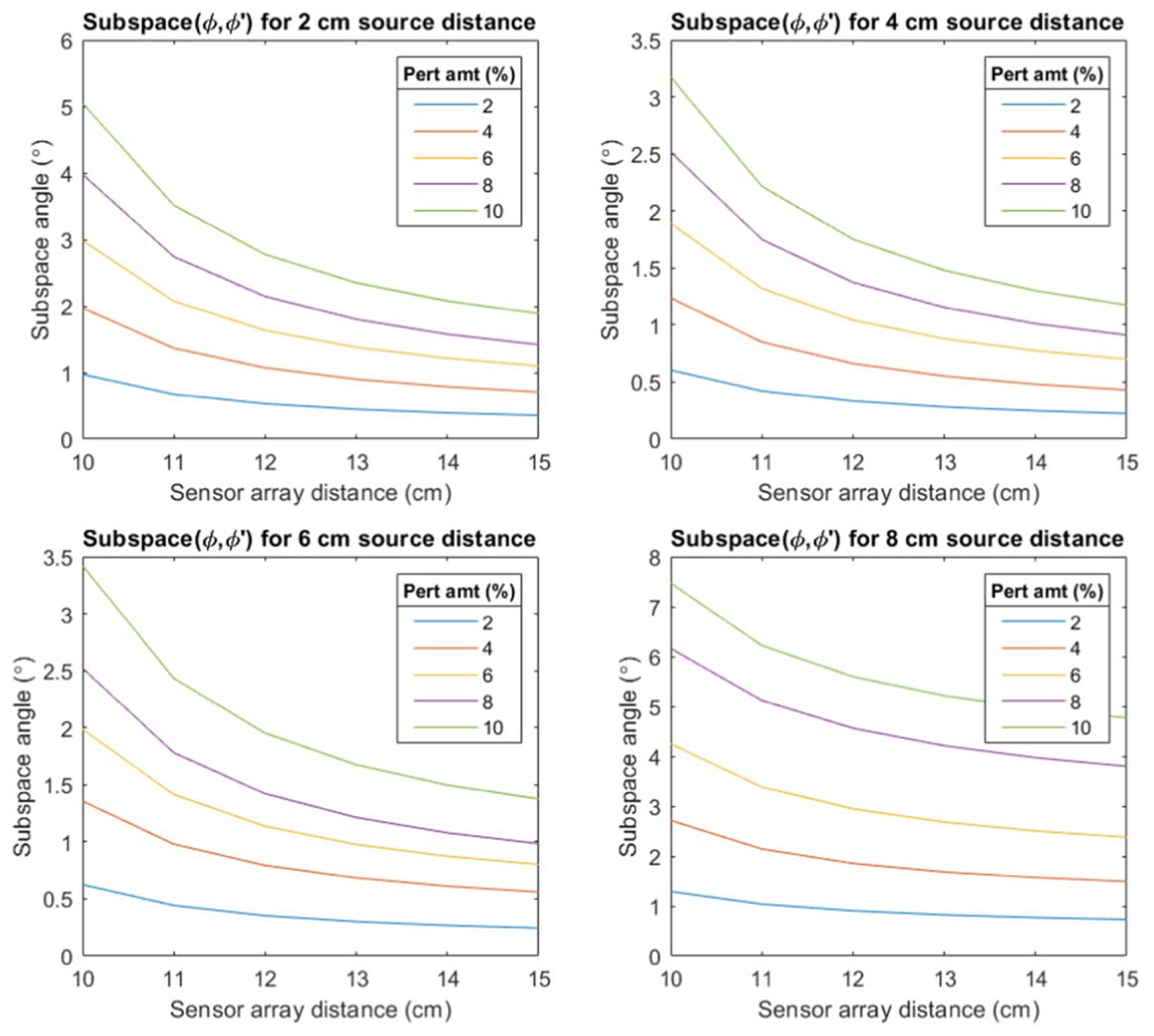
Plots of the subspace angle between the reference signal *ϕ* and perturbed signal *ϕ′* in the noiseless case as a function of sensor array distance for various source distances. The angle decreases for increased sensor array distances, thus the signal error caused by head model errors are less impactful for more distant sensor arrays. Moreover, as the perturbation of the head model increases, the subspace angle increases.

**Figure 3.**
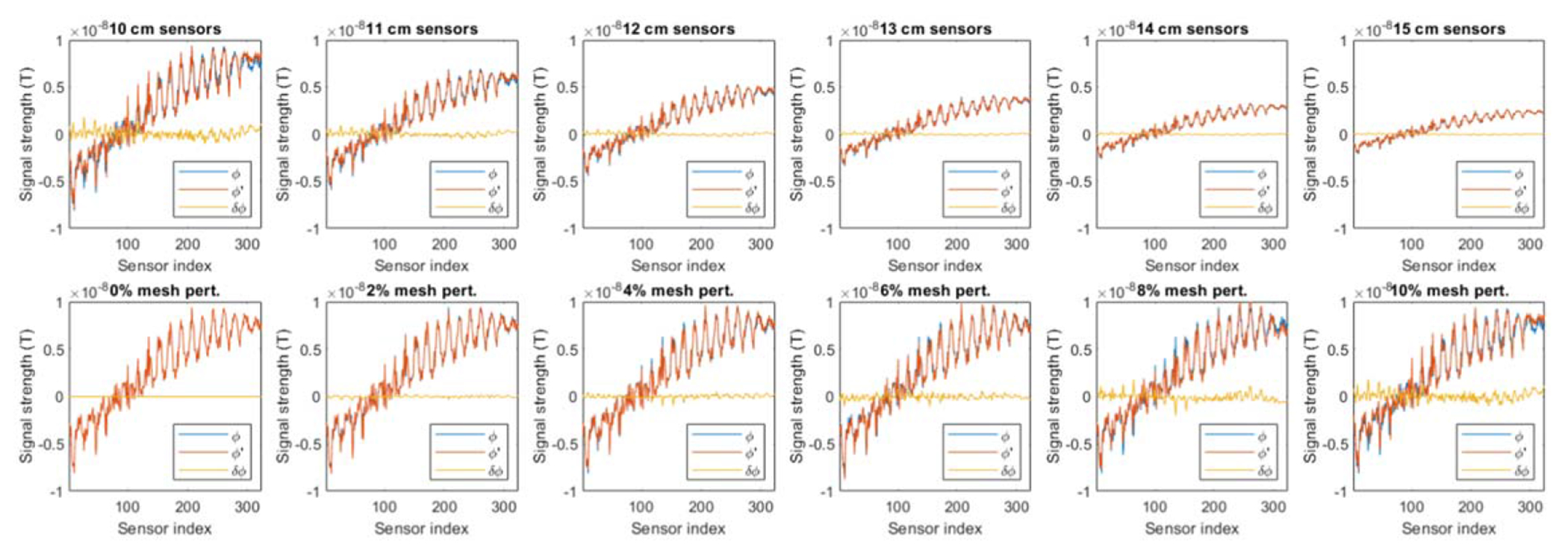
(First row) Plots of the signal vectors *ϕ* and *ϕ′* and signal error *δϕ* = *ϕ′* – *ϕ* for the 2 cm source with a 10% mesh perturbation across various sensor array distances. As sensor array distance increases, signal error decreases for the same amount of mesh perturbation. (Second row) Plots of *ϕ*, *ϕ′* and *δϕ* for the 2 cm source with a sensor array distance of 10 cm for various mesh perturbations. Increased mesh perturbations result in increased signal error.

First, we investigate how the error due to BEM mesh inaccuracy varies according to sensor array distance using equation (33). The sensor array radii were set to be from 10 cm to 15 cm, in increments of 1 cm (i.e. 1 cm to 6 cm from the surface of a 9 cm head model) and the signal was assumed the be noiseless. Figure 2 shows that for all the source distances considered, as sensor array distance increases, the subspace angle between the reference signal *ϕ* and perturbed signal *ϕ* decreases, indicating decreasing relative effects of mesh boundary inaccuracies as sensor array distance from the head increases. Moreover, smaller perturbations to the head model resulted in smaller subspace angles for a given sensor array distance when compared to higher perturbations as expected. These results may also be visually seen via plots of *ϕ* and *ϕ′* explicitly; we show this in figure 3 with the 2 cm source case across the various sensor distances and mesh perturbations for one of the 100 random mesh perturbation realizations. Also note the interesting observation from figure 2 that there is a ‘turnaround’ point in subspace angle as a function of source distance for a fixed mesh perturbation amount—from the 2 cm source case to the 4 cm and 6 cm source cases, the subspace angle decreases before increasing again in the 8 cm source case.

**Figure 4.**
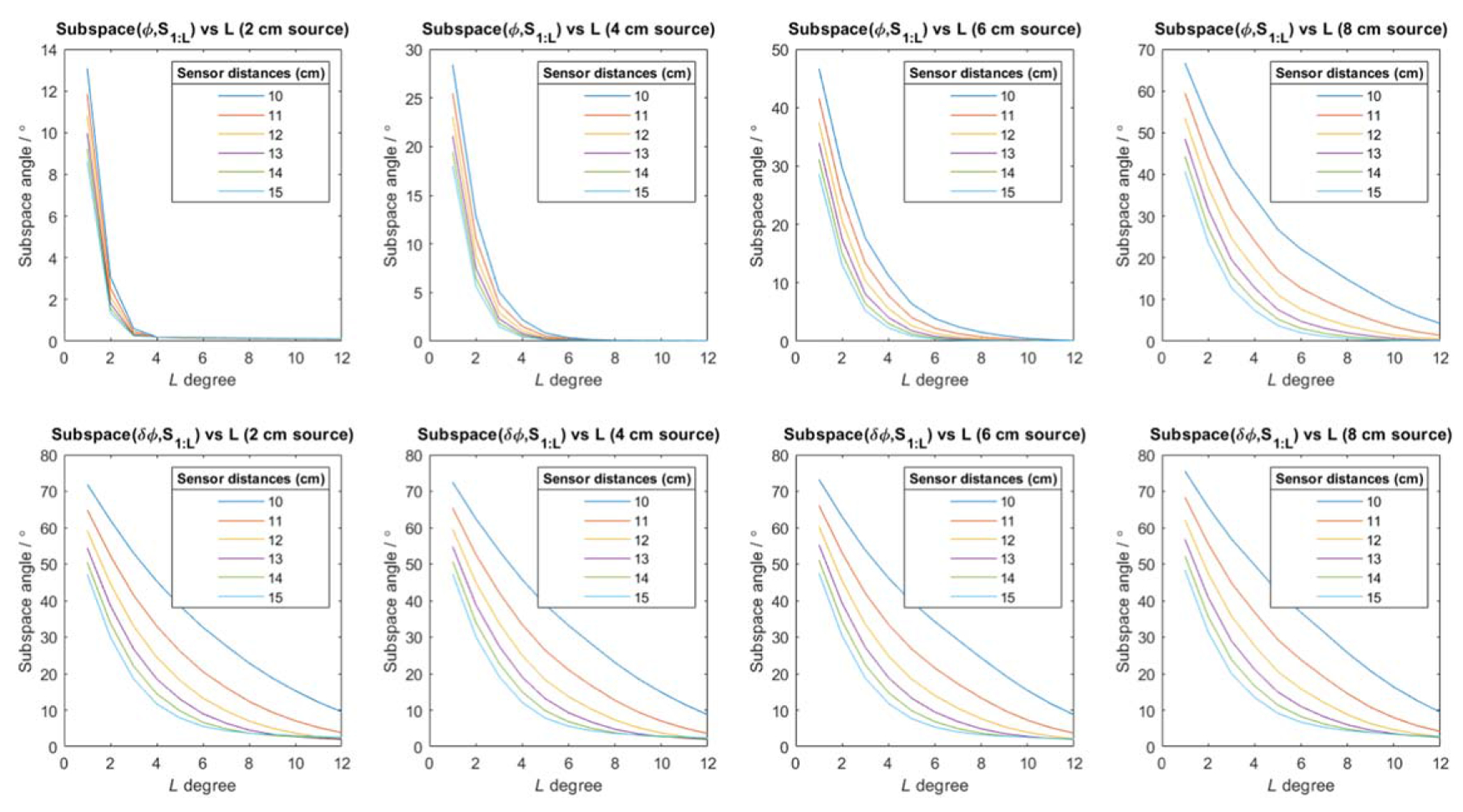
(Top row) Plots of the subspace angle between the total signal *ϕ* and the VSH basis matrix **S**_1:*L*_ for various source distances. *ϕ* can be explained well at a *l* = 12 degree truncation of **S**_1:*L*_ for all source distances. For closer sensor distances, the subspace angle between *ϕ* and **S**_1:*L*_ is higher for a given *l* degree truncation. (Bottom row) Plots of the subspace angle between the signal error *δϕ* and **S**_1:*L*_ for a 10% perturbed head model and various source distances. Similar to the top row, the subspace angle decreases for increasing sensor distances. However, the angles are higher than in the total signal case shown in the top row, indicating a higher spatial complexity of the signal and signal errors recorded by sensor arrays closer to the head.

Next, we determine if the additive error of the signal vector, *δϕ*, may be explained by increasing orders of *l* degree truncation, and if *δϕ* varies according to sensor distances. First, we show that the unperturbed signal may be explained to a large extent with *L* = 12 degree truncation for all the sensor array distances that we considered. This is shown in the first row of plots in figure 4; the subspace angle with *S*_1:*L*_ decays to become small (<5°) at *L* = 12 for all source distances. As expected, closer sensor array distances have higher subspace angles than further sensor arrays, which agrees with figure 2.

From the second row of plots in figure 4 however, we see that despite the total signal *ϕ* being well-explained at *L* = 12, the signal errors *δϕ* which consist mostly of higher frequency components require a higher *L* truncation to explain the signal for all source distances. The subspace angle still decreases for increased *l* degree truncation as expected (since *δϕ* = *δϕ_vol_* are still volume current contributions), and the subspace angles are smaller for increased sensor array distances. However, the angles are much higher than those of the total signal *ϕ*, indicating that higher spatial frequency components are affected more by inaccurate mesh modeling. This is because a higher l degree truncation required to fully explain the additive signal error *δϕ* of closer sensor arrays indicates that their *δϕ* has a more spatially complex pattern with higher spatial frequency components. This higher spatial complexity is due to the fact that higher spatial frequency components decay quickly with increasing distance, hence by placing sensors closer to the source, these components can be captured to give higher resolution of the signal error *δϕ*. Note that figure 4 is for the case where the vertices of the mesh was perturbed radially up to 10%. The plots for the different mesh perturbations are similar, hence we present just the 10% case here.

### 4.3. Source localization and orientation errors

From our results above which considers the noiseless signal case, the signal vector suffers from higher inaccuracies for closer sensor arrays due to the larger errors in their higher spatial frequency components. Deep and superficial sources also had higher signal inaccuracies due to a higher volume current contribution relative to primary currents. Here, we investigate if these observations will be seen in the form of source localization errors as well. The source localization procedure was done via a standard ECD fit using the ‘fit_dipole’ function in MNE-Python 1.0 (Gramfort et al 2013, 2014). The forward model here was computed using LC BEM however, as it is currently the only solving method implemented in MNE-Python.

**Figure 5.**
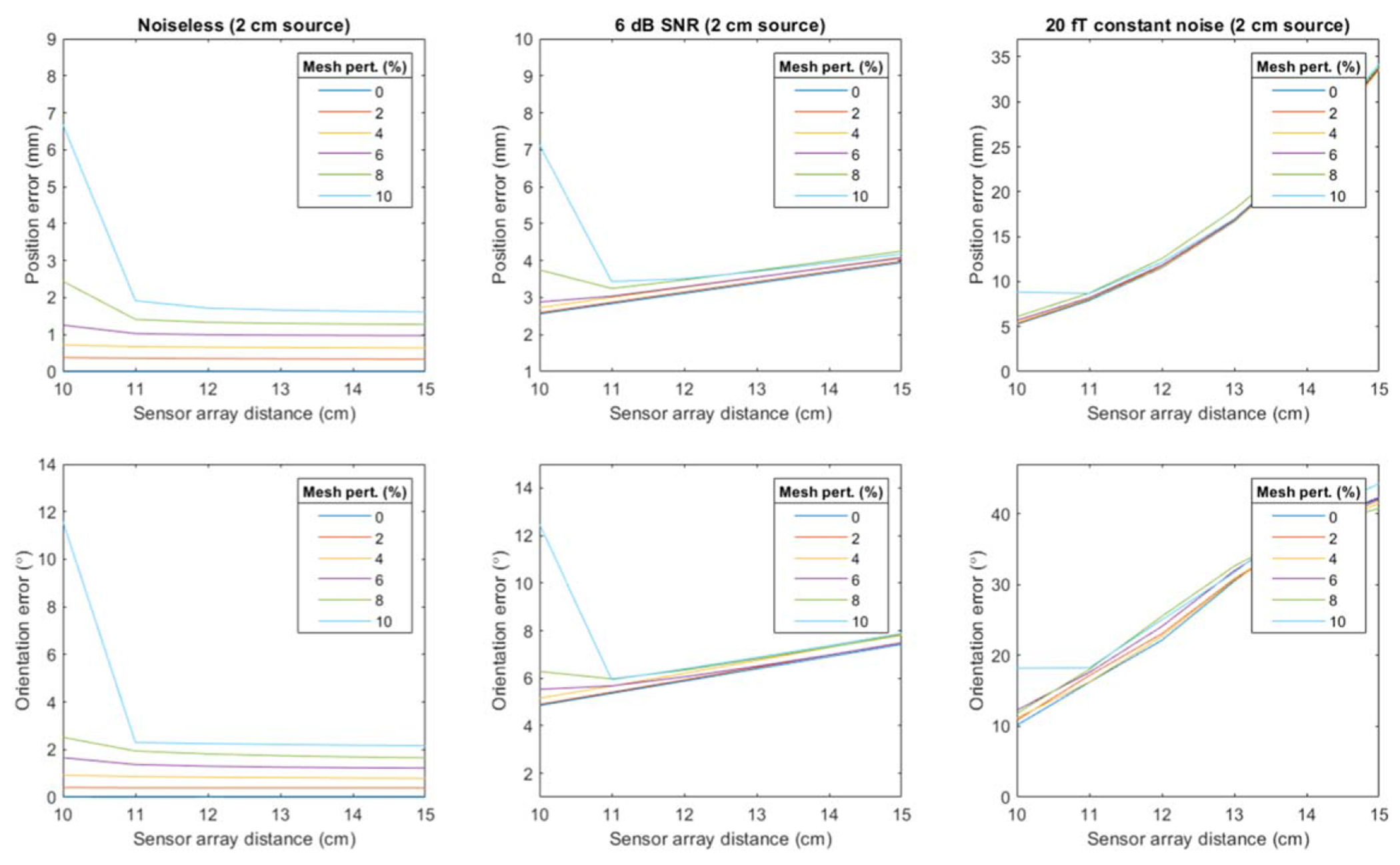
Plots of the position and orientation errors of the ECD fit for the 2 cm source case. (Left column) For noiseless signals, the errors increase for closer sensor array distances to the head. This agrees with the result that closer sensor array measurements give more inaccurate signals in the noiseless case. (Middle column) In the noisy case with an SNR level of 6 dB, the localization and orientation errors start to increase slightly as sensor array distances increase, indicating that the effects of better localization due to higher SNR is starting to balance out the effects of poorer localization due to more inaccurate signals captured by closer sensor array distances. (Right column) In the noisiest case with a constant noise level of 20 fT, the dipoles are localized more accurately at closer sensor distances, since the effect of higher SNR now outweighs the effects of signal error due to head model inaccuracies.

**Figure 6.**
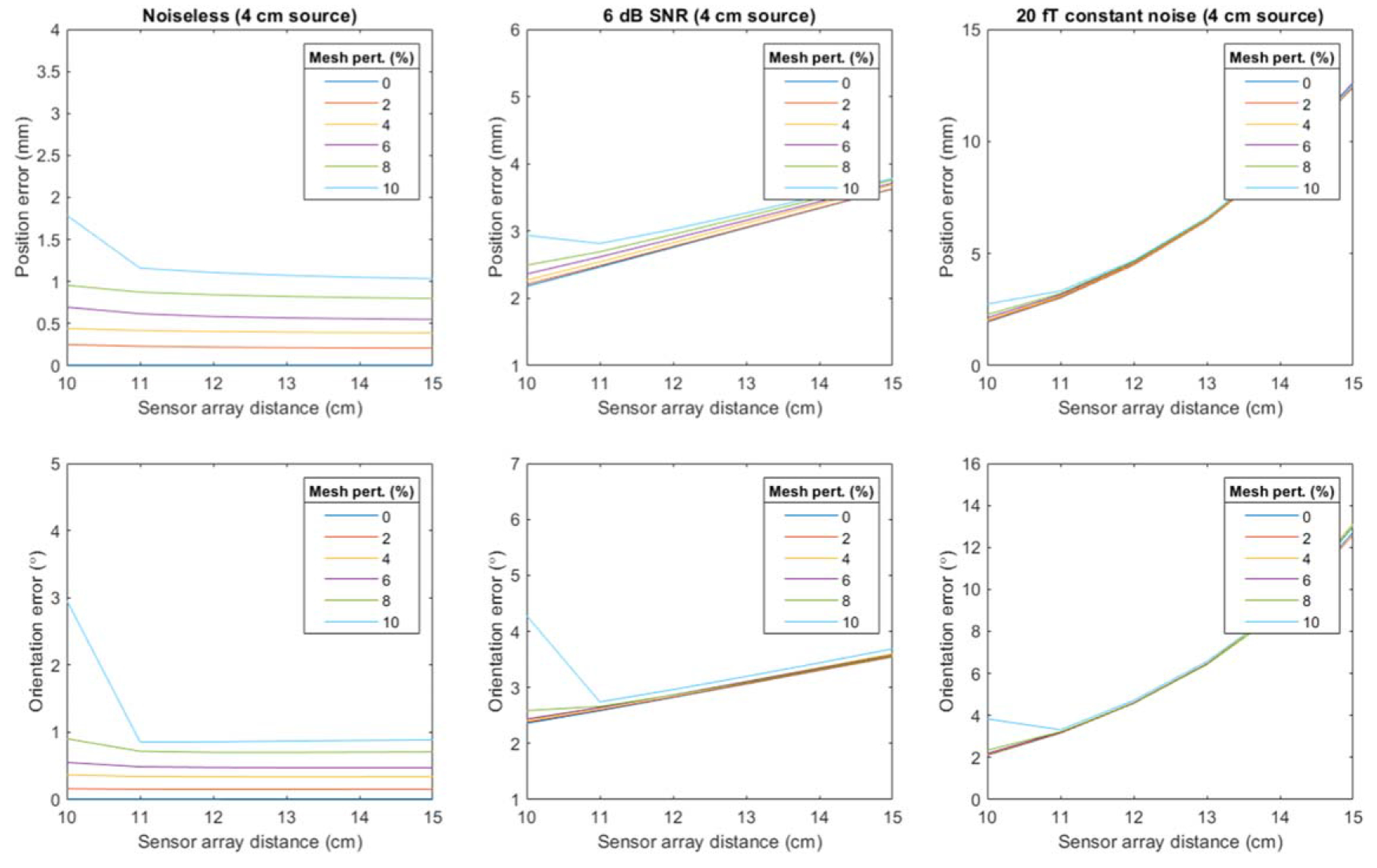
Plots of the position and orientation errors of the ECD fit for the 4 cm source case. The description of this figure is similar to figure 5.

**Figure 7.**
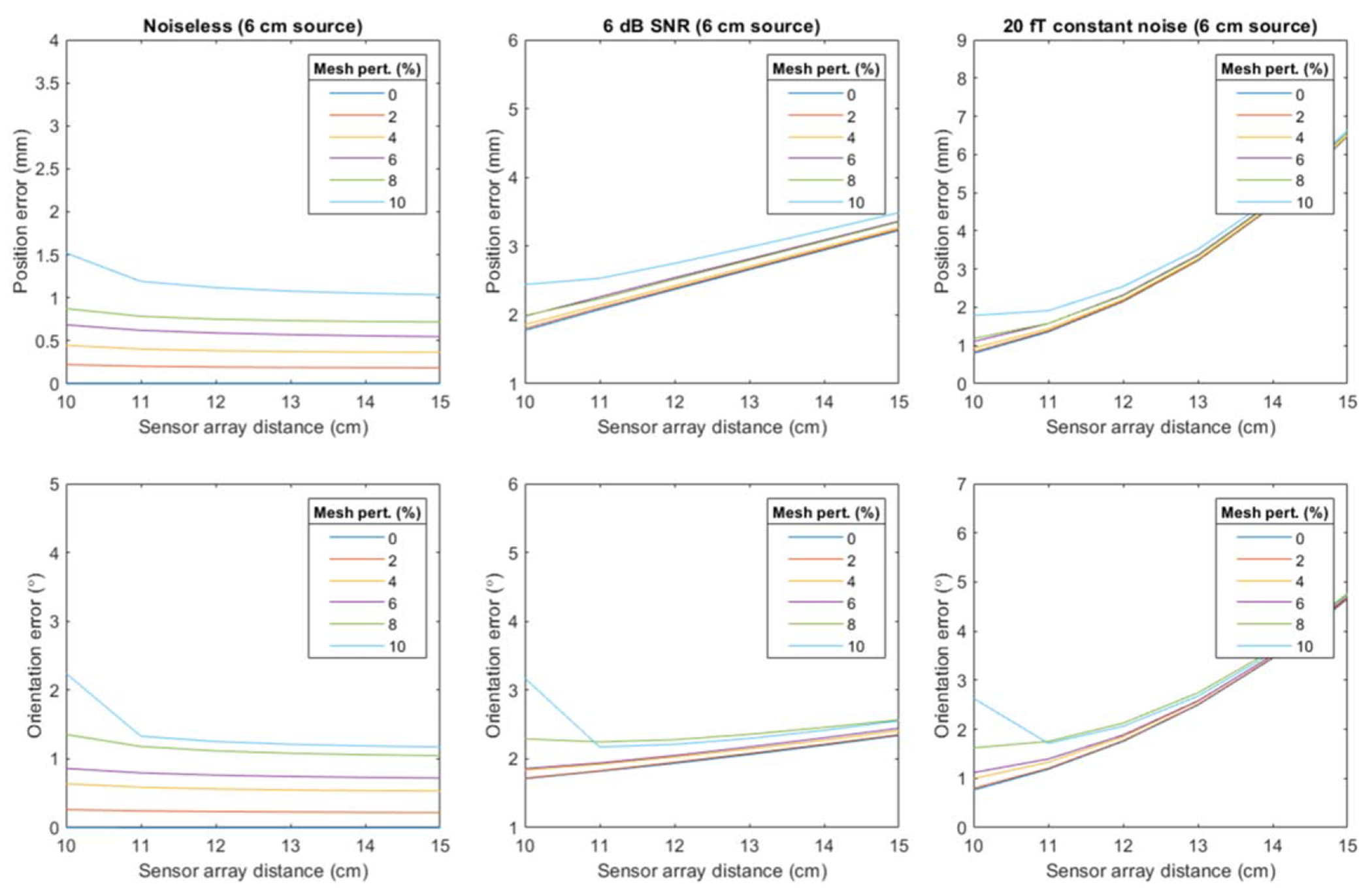
Plots of the position and orientation errors of the ECD fit for the 6 cm source case. The description of this figure is similar to figure 5.

**Figure 8.**
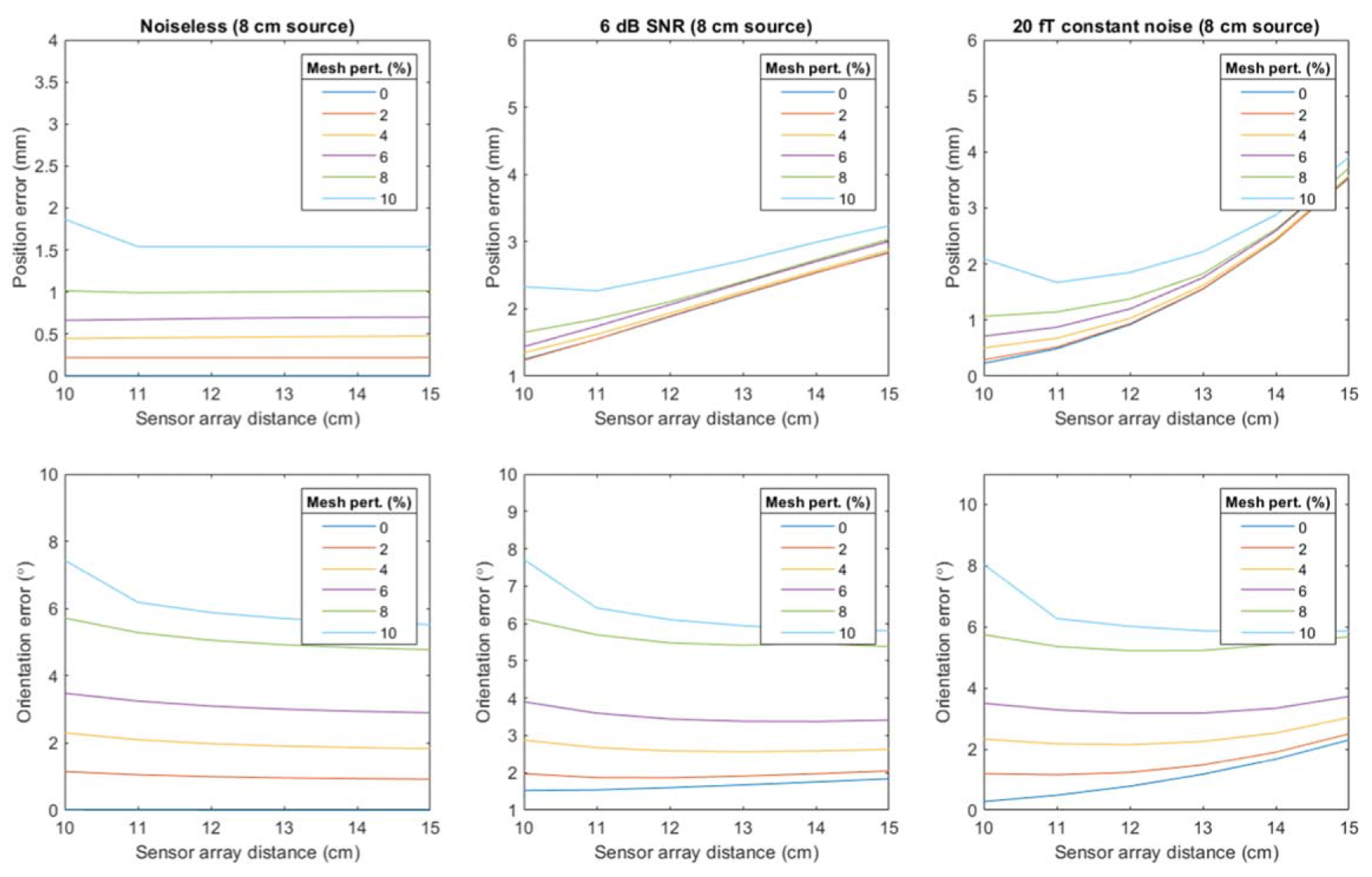
Plots of the position and orientation errors of the ECD fit for the 8 cm source case. The description of this figure is similar to figure 5.

The results for the noiseless case are shown in the first columns of figures 5, 6, 7 and 8, which correspond to the 2 cm, 4 cm, 6 cm and 8 cm source case respectively. Indeed, in the noiseless case, source localization and orientation errors are largest for closer sensor arrays, as well as deep and superficial sources for a fixed mesh perturbation. These observations are in agreement with the result for signal errors as presented above.

However, the primary motivation to move sensors closer to the head is because closer sensors have the potential to give more accurate source localization results due to higher SNR as mentioned in the introduction.

The definition of SNR (with units of decibels) used in MNE-Python 1.0 follows that of Goldenholz (Goldenholz et al 2008)

(38) $$\text{SNR}=10\;log\left\lfloor \frac{q^{2}}{N}\sum_{j=1}^{N}\frac{\phi_{j}^{2}}{s_{j}^{2}}\right\rfloor ,$$

where *q* is the source amplitude, *N* is the total number of sensors in the sensor array, *ϕ_j_* is the signal on sensor *j* for a source with unit amplitude, and $s_{j}^{2}$ is the noise variance on sensor *j*. We thus introduced various noise levels to determine the noise level at which the effect of having more accurate source localization due to higher SNR of closer sensors outweighs the effect of less accurate source localization due to the higher errors of the high frequency components of the noiseless signal. We found that this occurred at a SNR of around 6 dB (the sensor noise was varied to force a 6 dB SNR for a varying sensor array distances); the localization and orientation errors began to increase as sensor array distances increased at around an SNR of 6 dB. This is shown in the second column of figures 5, 6, 7 and 8. The rightmost column of figures 5, 6, 7 and 8 show that for an SNR greater than 6 dB, in this case a constant 20 fT noise level, then there is an improvement in source localization for closer sensor arrays. This means that a higher SNR allowing for better source localization resolutions outweighs the effects of BEM errors for noisy signals with SNR above approximately 6 dB.

## 5. Discussion

We note that there are many sources of errors for BEM, which may be broadly categorized as anatomical modeling errors and/or numerical errors. For example, the effect of conductivity errors on forward signals have been studied in (Haueisen et al 1997, Vallaghe and Clerc 2009, Stenroos and Hauk 2013, Stenroos and Nummenmaa 2016), and different mesh triangulation methods and basis choices to estimate the potentials within each triangle may result in numerical errors that affect accuracy of forward calculations (Meijs et al 1989, Schlitt et al 1995). Studies on head geometry errors, especially focusing on the skull, have also been conducted in for example (Chauveau et al 2004, Dannhauer et al 2011, Lanfer et al 2012). An overview of many of these errors can be found in Ferguson and Stroink (1997). In this paper, we have specifically investigated the effect of random vertex perturbations from an ideal spherical head model on the resulting signal. One other paper has explored head model errors from ideal spherical models as well, but with the assumption that the errors are sufficiently smooth so that they can be described with a spherical harmonic expansion accurately (Nolte et al 2001). Since our approach accounts for individual vertex perturbations, it offers more precise adjustments/corrections to discretized head models.

We acknowledge that a 1-shell spherical head model is unrealistic. However, this set up is simple and sufficient for the purpose of this paper—our goal is to investigate the errors of the signal in the spatial frequency domain and the source localization errors in a general sense due to inaccuracies relative to some reference model (in this case, a spherical head model). Moreover, a single layer allows for an arbitrary amount perturbation to be introduced to the vertices without the possibility of shells intersecting each other, hence permitting a more general proof-of-concept investigation. Multi-layered realistic head models as a reference will be considered for future work so that more apparently applicable results can be obtained.

As mentioned, the CC and LC BEM methods that we used are the most straightforward methods amongst all BEM methods. This does not necessarily mean that they are the most inaccurate in all scenarios, since their accuracy depends on numerous factors such as the error metric used, source distance, and head discretization method (Stenroos and Haueisen 2008). As a check, we verified our results with a denser head mesh triangulated with 2562 vertices. All the above observations hold, suggesting that our chosen mesh density with 642 vertices is sufficiently high to provide an accurate enough forward signal calculations.

## 6. Conclusion

In this paper, we have investigated the signal and source localization errors with respect to varying MEG sensor array distances due to inaccurate spherical BEM head geometries. This examination was motivated by next-generation OPM sensors that may be placed directly on the subject’s head. This results in signal measurements with higher spatial resolutions, including any inaccurate volume current contributions. As mentioned, for a piecewise homogeneous conductor model of the head, volume currents may be equivalently represented as surface currents at the head model boundaries. As such, head model inaccuracies lead to inaccurate volume current contributions.

We found that for signals with SNR greater than 6 dB, placing sensor arrays closer to the head causes higher relative signal errors for a perturbed spherical head model, and thus this leads to higher source localization errors. This is because the signal measured by these sensor arrays now contain finer spatial details due to the ability to now capture the fast-decaying high spatial frequency components of the magnetic field, and these high frequency components suffer from higher errors due to head model inaccuracies. Moreover, for deep and superficial sources, the signal and source localization errors are higher as well for a fixed mesh perturbation amount. For signals with SNR below 6 dB, the advantage of having higher SNR for closer sensor array distances outweighs the effects due to the increased errors arising from the high spatial frequency components, thus source localization errors decrease. This is consistent with the current understanding of the advantages of using OPM sensor arrays.

Our results from the consideration of a simple spherical head model indicate that for head models with errors, OPM sensor arrays likely result in more accurate source localizations for signals with noise. However, as signal noise levels decrease either via an improvement in sensor technology or signal processing methods (e.g. averaging over many trials), BEM head geometry errors may need to be minimized for sensors shifted closer to the head in order to avoid increased signal and source localization inaccuracies.

## Supplementary Material

Supp Material

Supplementary material for this article is available online

## Data Availability

No new data were created or analysed in this study. Data will be available from 31 January 2023.
