# Supplementary material for: Effects of head modeling errors on the spatial frequency representation of MEG
Source: Phys Med Biol. Author manuscript; Available in PMC 2026 Jul 15. (PMC13371167; doi:10.1088/1361-6560/accc06)
Supplement: Supp Material [file NIHMS2186477-supplement-Supp_Material.pdf]

Supplementary materials

Wan-Jin Yeo[1,2], Eric Larson[2], Joonas Iivanainen[3], Amir Borna[3], Jim McKay[4], Julia Stephen[5], Peter Schwindt[3], Samu Taulu[1,2]

[1] Department of Physics, University of Washington, Seattle, WA 98195, United States

[2] Institute for Learning and Brain Sciences, University of Washington, Seattle, WA 98195, United States

[3] Sandia National Laboratories, Albuquerque, NM 87123, United States

[4] Candoo Systems Inc., Port Coquitlam, BC V3C 5M2, Canada

[5] The Mind Research Network, Albuquerque, NM 87106, United States

April 21, 2023

1 Analytical first-order errors for CC BEM

From (24), we see that there are three terms that depend on the head model accuracy that affect the calculation of the magnetic field: the conductivities $\sigma_l^{\pm}$, the scalar electric potential $V(\mathbf{c}_l^m)$ (equivalently, the matrix $\mathbf{G}$), and the vector solid angle $\mathbf{\Omega}_l^m$. The latter two depend on the mesh accuracy, whereas the former depends on the conductivity values.

1.1 Perturbations to mesh vertices

First, we consider perturbations to V. By looking at the form of $\mathbf{G}$ as in (13), we see that there are two possible sources of error in the head model: the solid angle (i.e. vertex/triangle centroid perturbations), and the conductivity values of each region. Here, we offer analytical forms to compute the first-order errors due to each of these sources of error. Higher order corrections may be obtained with the help of e.g. Matlab or Mathematica.

The solid angle can be regarded as a scalar field in 12-dimensional space (3 coordinates per $\mathbf{r}$, $\mathbf{r}_1$, $\mathbf{r}_2$, $\mathbf{r}_3$). A perturbation to one of these 12 coordinates corresponds to perturbations in the x, y or z direction of one of the three vertices of the corresponding triangle $\mathbf{r}_1$, $\mathbf{r}_2$, $\mathbf{r}_3$, or the x, y or z direction of the point of reference $\mathbf{r}$. Let $\mathbf{r} = (x, y, z)$ and $\mathbf{r}_i = (x_i, y_i, z_i)$, where $i = 1, 2, 3$. For small perturbations, only the first-order expansion term is significant. If we let x be perturbed to become $x + \delta x$, the error in solid angle calculations (10), $\delta\Omega$, can be approximated as

$$\delta\Omega = \Omega\left(x + \delta x, y, z, x_1, \ldots, z_3\right) - \Omega\left(x, \ldots, z_3\right) \approx \left.\frac{\partial\Omega}{\partial x}\right|_{\mathbf{r},\mathbf{r}_1,\mathbf{r}_2,\mathbf{r}_3} \delta x \tag{1}$$

Let the argument within the arctan be $P = P(x, \ldots, z_3)$. The right hand side of (1) may be evaluated by the chain rule:

$$\frac{\partial\Omega}{\partial x} = \frac{\partial\Omega}{\partial P}\frac{\partial P}{\partial x} = \frac{2}{1+P^2}\frac{\partial P}{\partial x}. \tag{2}$$

Note that due to the symmetric form of the solid angle where the numerator obeys scalar triple product identity and the denominator has all terms that obey cyclic index permutations, we only need to evaluate 2 partial derivatives of P to get all 12 of them. Namely, we only need to evaluate one partial derivative with respect to any of the 3 coordinates of $\mathbf{r}$, and another with respect to any of the 9 coordinates of $\mathbf{r}_i$. Cyclic permutation of the coordinate indices $(x, y, z) \leftrightarrow (z, x, y) \leftrightarrow (y, z, x)$ then gives us the other partial derivatives with respect to the other 2 coordinates of the corresponding $\mathbf{r}$ or $\mathbf{r}_i$. Then, for the case of partial derivatives corresponding to coordinates of $\mathbf{r}_i$, permutation of vertex indices $(1, 2, 3) \leftrightarrow (2, 3, 1) \leftrightarrow (3, 1, 2)$ gives us the other 2 triangle vertices' 6 partial derivatives. Note that if we extend to higher-order partial derivatives, symmetry considerations may still be used to reduce the total number of partial derivatives to evaluate. However, mixed partials mean that more than 2 partial derivatives need to be evaluated necessarily.

One may also interpret the above 12 partial derivatives to evaluate as a 1-1 correspondence between the index permutations and 12 coordinates. The 3 $\mathbf{r}$ coordinates correspond to the 3 cyclic permutations on the

coordinate indices (x, y, z), whereas the 9 $\mathbf{r}_i$ coordinates correspond to the $3 \times 3 = 9$ possible pairings of the cyclic permutations of two sets of indices, namely the coordinate indices (x, y, z) and vertex indices $(1, 2, 3)$. If we denote the 3 coordinates of $\mathbf{r}$ as (x_1, x_2, x_3) and the 9 coordinates of $\mathbf{r}_i$ as x_j, $j = 4, \ldots, 12$, then the total perturbation of the solid angle is

$$\delta\Omega_l^m\left(\mathbf{c}_k^i\right) \approx \frac{2}{1+P^2}\left(\sum_{j=1}^{3} \left.\frac{\partial P}{\partial x_1}\right|_{\{\sigma_j^c\}} \delta x_j + \sum_{j=4}^{12} \left.\frac{\partial P}{\partial x_4}\right|_{\{\sigma_j^{c+v}\}} \delta x_j\right) \tag{3}$$

where $\sigma_j^c = (x_j, x_k, x_l)$ are the 3 possible coordinate index cyclic permutations, and σ_j^{c+v} are the 9 possible pairs of coordinate index and vertex index cyclic permutations. Any small perturbation of a vertex results in the adjacent triangles' perturbed centroids (i.e. perturbed observation points $\mathbf{r}$) and the adjacent triangles' change in solid angles; these two cases correspond to the first and second sums in (3) respectively.

The effects of the perturbations above may be represented by a sparse additive perturbative matrix $\delta\mathbf{G}$ as defined in (10), whose elements are calculated by (3) and (9). Note that the first sum of (3) corresponding to the case of perturbed centroids contribute to nonzero row entries, whereas the second sum corresponding to perturbed vertices contribute to nonzero column entries, due to our arrangement of the block elements in $\mathbf{G}$.

We now want to see how this affects $\mathbf{V}$. Let $\mathbf{A} \equiv (\mathbb{I} - \mathbf{G} + \mathbf{e}\mathbf{c}^T)$ and $\tilde{\mathbf{A}} \equiv \mathbf{A} + \delta\mathbf{G}$. If $\tilde{\mathbf{A}}$ is non-singular, then

$$\tilde{\mathbf{A}}\tilde{\mathbf{A}}^{-1} = (\mathbf{A} + \delta\mathbf{G})\,\tilde{\mathbf{A}}^{-1} = I \tag{4}$$

$$\implies \mathbf{A}^{-1} = \left(I + \mathbf{A}^{-1}\delta\mathbf{G}\right)\tilde{\mathbf{A}}^{-1} \tag{5}$$

$$\implies \tilde{\mathbf{A}}^{-1} - \mathbf{A}^{-1} = -\mathbf{A}^{-1}\delta\mathbf{G}\,(\mathbf{A} + \delta\mathbf{G})^{-1} \tag{6}$$

Therefore, errors in potential are given by

$$\delta\mathbf{V} = -\mathbf{A}^{-1}\delta\mathbf{G}\,(\mathbf{A} + \delta\mathbf{G})^{-1}\,\mathbf{V}_\infty. \tag{7}$$

Next, we consider the first-order perturbation to the vector solid angle (26). It depends only on the coordinates of the triangles' vertices and may be obtained in a straightforward manner,

$$\delta\boldsymbol{\Omega}_l^m \approx \sum_{i=1}^{3}\left[\sum_{j=1}^{9} \frac{\partial\left(\gamma_{i-1} - \gamma_i\right)}{\partial x_j}\delta x_j\right]\mathbf{r}_i. \tag{8}$$

1.2 Perturbations to conductivity

We now consider perturbations to $\sigma^{\pm}$, which are conductivity values within the layers of the head model. This is an easier case to deal with, since we may simply add a perturbative constant $\delta\sigma$ to each σ. For the conductivity term within $\mathbf{G}$, let us denote this error term as

$$\begin{aligned}\delta\sigma_{k,l} &= \frac{\sigma_l^- + \delta\sigma_l^- - \sigma_l^+ - \delta\sigma_l^+}{\sigma_k^- + \delta\sigma_k^- + \sigma_k^+ + \delta\sigma_k^+} - \frac{\sigma_l^- - \sigma_l^+}{\sigma_k^- + \sigma_k^+} \\ &= -\frac{\left(\sigma_l^- - \sigma_l^+\right)\left(\delta\sigma_k^- + \delta\sigma_k^+\right) + \left(\sigma_k^- + \sigma_k^+\right)\left(\delta\sigma_l^+ - \delta\sigma_l^-\right)}{\left(\sigma_k^- + \sigma_k^+\right)^2 + \left(\sigma_k^- + \sigma_k^+\right)\left(\delta\sigma_k^- + \delta\sigma_k^+\right)}\end{aligned} \tag{9}$$

Together with (3), the total perturbative matrix $\delta\mathbf{G}$ for small vertex perturbations and arbitrary conductivity inaccuracies is

$$\delta G_{k,l}^{i,m} = -\frac{1}{2\pi}\delta\sigma_{k,l}\delta\Omega_l^m\left(\mathbf{c}_k^i\right). \tag{10}$$

The errors in the total magnetic field up to first order with respect to perturbations of the components of $\mathbf{r}_i$ as well as conductivities σ may be given by

$$\delta\mathbf{B}\left(\mathbf{r}\right) \approx \frac{\mu_0}{4\pi}\left\{\sum_{l=1}^{N_S}\left(\sigma_l^- - \sigma_l^+\right)\sum_{m=1}^{N_l}\left[\delta V\left(\mathbf{c}_l^m\right)\mathbf{\Omega}_l^m + V\left(\mathbf{c}_l^m\right)\delta\mathbf{\Omega}_l^m\right]\right.$$
$$\left. + \sum_{l=1}^{N_S}\left(\delta\sigma_l^- - \delta\sigma_l^+\right)\sum_{m=1}^{N_l}V\left(\mathbf{c}_l^m\right)\mathbf{\Omega}_l^m\right\} \tag{11}$$

with its terms given by (7), (10), (9), (3), and (8).
